# The Late Dr. James Johnson

**Published:** 1846-02

**Authors:** 


*The Late Dr. James Johnson.*—It would be treason to the Literature of Medicine, and a wrong to the memory of an able physician, to suffer the death of Dr. James Johnson to pass without some notice. If the pursuit of knowledge under difficulties is honorable—if its attainment by incessant industry is laudable—if the example of a man rising without friends, or family, or wealth, to a high place in his profession, and to general estimation with the public, is calculated to be useful to those whose career is yet to be run—then a sketch of the life of Dr. James Johnson will not be without its value. We regret that our space is too limited to admit of such ample details as we could wish; but that regret is diminished by the conviction that the present editors of the "Medico-Chirurgical Review" will supply our deficiencies.

James Johnson, or, rather, Johnston, for such was really his name, was the youngest son of a family of Scotch extraction, settled on the banks of Lough Neagh, in the county of Derry, in Ireland. Like Codden, he might boast that he was a "farmer's son." Born in February, 1777, and dying on the 10th of October, 1845, he was in his 69th year at the time of his decease. His early education, such as it was, he obtained at a grammar-school, kept by a Catholic pedagogue, the brother of the parish priest. The village school-boy was the type of the future man; for he confessed that he was miserable when not at the head of his class, and would sit up till midnight conning the lessons of next day. At the age of fifteen, this instruction, whatever its amount, was at an end, and we may readily suppose that it formed a small portion of that varied, extensive, and miscellaneous information which distinguished him in after life. He was now apprenticed to a surgeon-apothecary in the county of Antrim, whence he was transferred to another in Belfast, and, at the end of four years, went to London, without either money or friends. The manner in whieh he contrived to obtain an acquaintance with anatomy and surgery, at long intervals and by brief instalments, might shame the sybaritic students of our day In 1798, he passed an examination as surgeon's mate, in the Navy, and was appointed to the Mercury frigate, where he devoted every hour to study, visiting the naval hospitals whenever the ship was in harbor, and winning the golden opinions of his captain, who winked at his absence from the ship for some months in the winter of 1799, when he worked night and day in London. At the age of 22, he was made full surgeon in the Navy, and accompanied the expedition to Egypt. His fatigues and exertions produced an illness which compelled him to return to London, where he studied in Great Windmill Street, under Mr. Wilson, who stated in a certificate, that he actually *lived* in the dissecting room. He had expended his last guinea, and midwifery lectures were yet to be obtained. He applied to Dr. John Clarke, who, with characteristic generosity, instantly gave him a ticket of admission, and invited him to his table.

In 1803, he sailed for the East, and during the next three years, in India and in China, he laid the foundation for his first, and perhaps, most permanent work, "The Influence of Tropical Climates on European Constitutions." Of that work, it is only necessary to observe, that it is distinguished for soundness of physiological views, acuteness of observation, and variety of matter. It is still the text-book of the tropical practitioner, and is likely long to continue so. In autumn 1806, he married Miss Charlotte Wolfenden, of Lambeth, who now survives him, and by whom he has had six children. The eldest and the youngest have followed the profession of their father; the former, Mr. Henry James Johnson, being assistant-surgeon to St. George's Hospital, and the latter, Mr. Alliot Johnson, residing there as house-surgeon. The work on Tropical Climates was not published till 1812, and then at his own risk and expense. Its success was decisive, though not immediate. In 1809, he was at Walcheren, and this and his Indian expedition sapped the vigour of a constitution naturally excellent; for, in the East, he suffered from dysentery, which was destined to cut him off at Brighton, after the lapse of 40 years,—and at Walcheren, he contracted ague, which, as has been the case in many instances, re-appeared in London, and nearly proved fatal to him there.

At the peace of 1814, Dr. Johnson served in the "Impregnable," when the late King William IV., then Duke of Clarence, hoisted his flag, for the purpose of conveying the Emperor of Russia, King of Prussia, &c., to this country. He attended his Royal Highness during an attack of "hay fever," and so pleased was the Duke with him, that he made him his surgeon-in-ordinary, appointed him "physician extraordinary" on his accession, and always treated him with a considerate kindness.

At the conclusion of the war, Mr. Johnson settled at Portsmouth, as a general practitioner, and was getting into good practice, when ambition and ill health both prompted him to try his fortune in London. He had taken out a Scotch degree, possessed about five hundred pounds, had one friend in the metropolis, Sir Wm. Young, and with this stock of worldly means he committed himself, at the age of 41, with a wife and five children, to the mercies of the modern Babylon. Though weak in body, of nervous temperament, and desponding turn, he had that determination and singleness of purpose which discards "impossible" from its vocabulary; like Sheridan, he probably felt "it was in him, and must come out."

It was a bold step for a moneyless and a friendless man to start in London:—it was bolder, at his own risk and expense, to originate at the same moment the "Medico-Chirurgical Review." But its success was at once the omen and means of his own, and no quarterly medical journal has ever attained a larger circulation, or exerted wider influence. His labors for a long period were arduous—practice in the day was succeeded by the toils of the desk at night; and for fifteen years he wrote every page of a work of considerable bulk, and remarkable for the condensation of its matter. This could not go on for ever. Hæmorrhoids and fistula, for which he was operated on by the late Sir Astley Cooper, and the present Mr. Guthrie, were followed by dyspepsia, in such an aggravated form as those only can understand who know what the malady is to a nervous frame, exhausted by bodily and mental labor. But out of evil comes good. His own sufferings directed his attention to the subject of Indigestion, and were the immediate cause and indeed foundation of his essay upon that complaint. The publication of that work at once raised his practice to as high a pitch as was compatible with his own inclinations and strength. The remainder of his life was as active, though not as chequered and anxious as that which had gone before. Incessantly occupied with his patients, the Review, his various works, (for he was an author to the last,) and his tours of health, in which he engaged with all the ardour of a boy, he could scarcely have been said, except in sleep, to have passed an unoccupied hour. His meals were hurried, he entered into no society, and his life would not seem to have much of enjoyment in it. But it suited his tastes, and was the natural fruit of that restless energy which had made him all that he was. A mere enumeration of his published works, none of them compilations, will be sufficient evidence of his industry, some of them, at least, of his talent as a writer. The list will be found below.

It only remains to weigh, in a few words, his public and private character. The latter is easily disposed of,—a good husband, a kind father, a warm-hearted generous man, his memory is embalmed in the affectionate remembrances of those who were connected with him. As an author, he was remarkable for facility of composition, a felicitious, though not always a correct style, and an original vigour and raciness of observation and expression that redeem some faults, and make his works eminently readable. He may almost be called the Cobbett of Medical Literature, the same boldness, terseness, and straightforwardness being characteristic of the writings of both. The popular seal of success has at all events been set on all he wrote, whether professional or general. Every production of his pen has met with a large sale, and most of them have gone through numerous editions.

As a practitioner, he was, in many respects, a model. Simple, unostentatious, his kindness was proverbial, and probably no man, since Dr. Baillie, has been more beloved by his patients. His liberality amounted to a fault, (being oftentimes imposed on) and almost indiscriminate. It has curtailed what might have been a large private fortune, but it has achieved its object, for it gratified a kindly disposition, and ministered to the necessities of others. Peace to his ashes, for they are those of an able, and what, perhaps, is better, of a good man.

WORKS BY DR. JAMES JOHNSON.

Tour in Ireland, with Meditations and Reflections.

Influence of Tropical Climates on European Constitutions; with additions by J. Martin, late Presidency Surgeon, and Surgeon to the Naval Hospital, Calcutta.

Practical Researches on Gout.

An Essay on Indigestion.

Change of Air, or the Pursuit of Health and Recreation.

Influence of Civic Life on Human Health.

On Diseases of the Liver.

The Recess, or Relaxations in the Highlands and Lowlands.

Economy of Health, or The Stream of Human Life from the Cradle to the Grave.

Pilgrimages to the German Spas.

Excursions to the Principal Mineral Waters of England.

The Medico-Chirurgical Review.—[*London and Edinburgh Monthty Journal.*

